# Image-based and ML-driven analysis for assessing blueberry fruit quality

**DOI:** 10.1016/j.heliyon.2025.e42288

**Published:** 2025-01-27

**Authors:** Marcelo Rodrigues Barbosa Júnior, Regimar Garcia dos Santos, Lucas de Azevedo Sales, Rônega Boa Sorte Vargas, Angelos Deltsidis, Luan Pereira de Oliveira

**Affiliations:** Department of Horticulture, University of Georgia, Tifton, GA, 31793, USA

**Keywords:** Predictive models, Artificial intelligence, RGB images, Sugar content, Fruit firmness, Precision horticulture

## Abstract

The assessment of blueberry fruit quality is traditionally conducted through laboratory equipment. Despite its high accuracy, this method remains destructive, labor-intensive, time-consuming, and costly. Consequently, there is a pressing need for innovative solutions such as image-based and artificial intelligence (AI)-driven analysis. To address these limitations, this study aimed to analyze whether an approach based on mobile image-based analysis combined with machine learning (ML) algorithms could develop a non-destructive framework for evaluating blueberry fruit quality, specifically focusing on total soluble solids (TSS) and firmness. Firstly, we collected numerous blueberry samples during the maturity stage to construct a comprehensive dataset. These samples were meticulously analyzed in a laboratory for diameter, TSS, firmness, and color. Simultaneously, RGB images were captured using a mobile device. These images were processed to extract spectral bands (red, green, and blue). Eight ML algorithms were employed to develop predictive models capable of predicting the qualitative parameters of the blueberries. Initially, correlation analysis demonstrated that RGB images suggestively contribute to fruit quality assessment (r < 0.41). However, the integration of ML algorithms significantly enhanced the predictive accuracy of these models (R^2^ = 0.71–0.99, MAE = 0.003–0.28, and RMSE = 0.004–0.31). This innovative approach differs from existing methods by providing a non-destructive, cost-effective, and efficient alternative to assess blueberry fruit quality. Our findings, therefore, confirm the applicability of mobile images for supporting high-quality blueberry harvesting. These advancements can potentially reduce labor costs, increase yield quality, and support advancements in precision agriculture.

## Highlights

• A total of 3600 blueberry fruits were analyzed for quality using advanced image analysis.
• Non-destructive predictions of TSS and firmness were achieved through RGB mobile imaging.
• Eight machine learning models were implemented to accurately predict fruit quality.
• This approach enables further smart harvest decisions, ensuring the selection of high-quality blueberries.

## Graphical abstract

**Figure:**
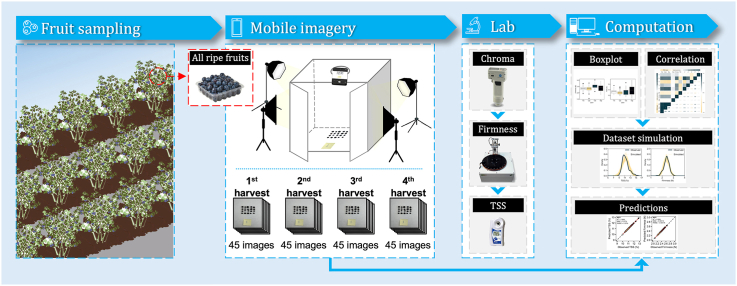

## 1 Introduction

Blueberry (*Vaccinium corymbosum* L.) is a high-value specialty crop extensively cultivated across various regions, particularly in North America, Europe, and parts of Asia [1]. Annually, these regions collectively produce nearly 655 thousand tons of blueberries, contributing substantially to both local and global economies [2]. The species is highly regarded in agriculture for its economic profitability and exceptional nutritional profile, characterized by high concentrations of antioxidants, vitamins, and dietary fiber [3]. These bioactive compounds have been associated with various health benefits, including the potential reduction in the risk of chronic diseases, positioning blueberries as a pivotal crop in the functional food sector [4]. The cultivation of blueberries varies by region, with different varieties adapted to specific climatic conditions, particularly in terms of cold hardiness and chill hour requirements [5]. In the United States, the predominant type is the highbush blueberry, which is further classified into northern (NHB) and southern (SHB) varieties [6]. SHB cultivation, in particular, involves the establishment of plants in the field, with plantations reaching peak commercial yields in less than 4 years [7,8]. Naturally, a mature SHB plant can remain productive for 10–20 years under optimal conditions [9].

Blueberry plants enter their harvest season annually, typically lasting about one month. During this period, fruit is usually harvested on a weekly basis and subsequently distributed for either fresh market consumption or industrial processing [10]. Traditionally, harvest timing is determined by visual inspection of fruit color, with blueberries considered mature once they turn dark blue [11]. However, this method relies on human visual assessment to determine ripeness, making the process labor-intensive, subjective, and not easily scalable. Moreover, visual assessments do not account for critical qualitative parameters such as sweetness and firmness, which are key indicators of fruit quality. While sweetness represents the accumulation of glucose, sucrose, and total sugars [12], firmness is an important parameter for fresh fruits as it influences the consumer's preferences and postharvest storage potential [13]. Despite their importance, traditional quality analyses to determine optimal harvest time are seldom performed, as they require destructive laboratory tests that are costly, time-consuming, and impractical for on-the-spot decision-making. This lack of quality assessment further highlights the need for technological advancements that can provide immediate and accurate measurements of key quality parameters, such as sweetness and firmness. Addressing these gaps is essential to enhancing both the efficiency and quality of blueberry harvesting. Furthermore, this approach would integrate blueberry crops into the precision agriculture framework by systematically analyzing and understanding the variability of these quality parameters across the field.

A thorough review of the scientific literature shows substantial research contributions in the analysis of blueberry fruit quality, predominantly through image-based techniques. For instance, Li et al. [14] and Tan et al. [15] conducted studies focused on identifying blueberry fruit at various growth stages using natural outdoor images. Their methodology successfully classified mature and near-mature fruits, as well as near-young and young fruits, achieving accuracy rates exceeding 85 %. Similarly, MacEachern et al. [16] employed convolutional neural networks (CNNs) to detect blueberry fruits at different maturity stages and estimate yield. Their models, particularly those developed using you only look once (YOLO)v4, demonstrated high accuracy in predicting green, red, blue, and ripe berries, with mean average precisions (mAP) of 79.79 % and 88.12 %, respectively. Xiao et al. [17] further advanced this field by utilizing YOLOv5 to detect blueberry fruit maturity, achieving an average recall of 92.0 % and a mAP of 91.5 % at a 0.5 threshold. Despite the promising results of these studies, they are limited by their reliance on visual classification for ground truthing, introducing a level of subjectivity. While visually ripe fruits may appear similar, subtle color variations that impact quality may be undetectable to the naked eye. Furthermore, these studies did not incorporate comprehensive quality assessments, such as measurements of sweetness or firmness. Conversely, Park et al. [18,19] explored the use of hyperspectral microscope images combined with deep learning (DL) to determine blueberry fruit firmness. Although their approach showed potential with accuracy rates exceeding 85 %, it remains limited by the need for laboratory-based equipment, which is time-consuming and costly. Mu et al. [20], on the other hand, proposed a method to detect the blueberry fruit quality using a professional camera and DL algorithms. They collected blueberry fruits at seven different stages of maturity and analyzed skin pigments, total acid, and total sugar. For data analysis, they used the visual geometry group network (VggNet). Although their results achieved high precision (R^2^ = 0.93–0.97), they did not focus on firmness, an important quality component. Additionally, the authors highlighted that the developed model required a large amount of memory to use on an embedded platform. Notably, there has been a lack of research specifically targeting the quality attributes of blueberry fruits. This gap emphasizes the pressing need for novel studies that focus on the estimation of sweetness and firmness concentrations in blueberry fruits. Moreover, while previous studies have demonstrated high accuracy in DL techniques, they necessitate significant computational power, making them costly. In contrast, machine learning (ML) offers a more suitable approach for such studies. One of the key advantages of ML models over DL approaches lies in their interpretability and computational efficiency. ML algorithms often require less computational power and can be more easily tuned and interpreted, making them well-suited for problems involving smaller datasets and limited computational resources.

Therefore, in this study, we aimed to analyze whether a mobile image-based approach combined with ML algorithms could assess the qualitative parameters of blueberry fruits. Initially, we captured images of blueberry fruits and subsequently analyzed whether the spectral information derived from these images could serve as a viable input for developing predictive models for total soluble solids (TSS) and firmness. This approach presents a significant opportunity to broaden the field of precision agriculture. By enabling more accurate predictions of fruit quality, our approach supports the ability to selectively harvest only high-quality fruit, contributing to reducing waste and optimizing the use of resources and, consequently, the overall sustainability of the agricultural supply chain.

## 2 Material and methods

### 2.1 Blueberry fruit sampling

Field sampling was conducted in a commercial blueberry field near Homerville, GA, USA (30°50′43"N, 82°40′06"W). In this orchard, seven-year-old plants Farthing (Southern highbush) were selected for this study. We aligned our sampling dates with the farmer's harvest schedule, which occurred four times during the season from April 23 to May 23, 2024. For the sampling process, 45 representative plants were selected. During each sampling event, all visually ripe fruit (dark blue color) from the designated plants were manually harvested and placed into vented clamshell containers. The harvested fruits were then stored in a cooler to maintain freshness before being transported to the Postharvest Physiology Laboratory in Tifton, GA, for quality analysis. Twenty fruits from each plant were randomly selected for laboratory and image analysis, resulting in a total of 180 samples being collected (45 plants × 4 data collection dates).

### 2.2 Image acquisition

In this study, we utilized a mobile device (Apple iPhone 15 Pro Max, Cupertino, CA, USA) to capture images of the blueberry fruits. The device is equipped with three rear cameras: a main 48 MP RGB camera with a 24 mm focal length and an f/1.78 aperture, a second 12 MP ultra-wide camera with a 13 mm focal length, an f/2.2 aperture, and a 120° field of view, and a third 12 MP 2x telephoto camera (enabled by a quad-pixel sensor) with a 48 mm focal length and an f/1.78 aperture. For this research, we employed the main camera in Raw max mode, using 2x zoom, exposure time of 1/980 s, and a square format (1:1), guaranteeing images of 886x886 pixels and a resolution of 72 dpi. The images were saved in .JPEG format with approximately 156 kilobytes in size. All images were captured at a height of 50 cm from the samples using an overhead perspective. To maintain consistent conditions, we placed the samples in a photographic studio with a white matte solid background and standardized artificial lighting. Each sample was tagged with an ID number for later identification and stored on a computer to prevent mistakes and misidentification. Additionally, all images were taken by the same individual using the same device, ensuring no interference from environmental degradation or noise.

### 2.3 Image processing

**Fig. 1:**
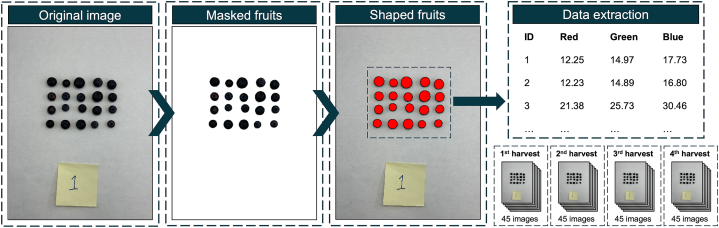
Overview of image processing workflow. The process begins with the upload of the original image. A mask was then applied to remove the background, followed by shaping the fruits to facilitate data extraction. A total of 180 images were processed in this study.

All 180 sample images (3600 individual berries) underwent processing which involved background removal, fruit recognition, and spectral information extraction (Fig. 1). The entire procedure was performed using the R programming language (version 4.4.0) through the RStudio interface (version 2024.04.2 + 764), primarily employing the “FIELDimageR” [21] and “FIELDimageR.Extra” [22]. Initially, the images were loaded, and background removal was conducted. For this step, the Brightness Index (BI) [23] was used to create a mask with the *fieldMask* function. A threshold of 85 BI was meticulously defined to exclude background information with values lower than this threshold. This value was carefully chosen to effectively remove the background without eliminating any fruit information, ensuring complete data retention. Additionally, the mask generated a polygon encompassing each object. Subsequently, these polygons representing each blueberry fruit were utilized for spectral data extraction. The spectral information was separately stored in a data frame with columns for Red, Green, and Blue values. For each 20-fruit sample, the spectral data was averaged, resulting in a single representative value for each spectral band. Furthermore, the ID number and data collection date were meticulously added to the data frame for later identification and analysis. This combined data was subsequently integrated with the laboratory data for comprehensive analysis.

### 2.4 Laboratory analysis

Following each imaging procedure, the 180 samples, each consisting of 20 blueberries, were submitted for quality analysis. Initially, the diameter of the blueberries was measured using a digital caliper (iGaging, San Clemente, CA, USA). Subsequently, a color analysis was conducted using a chroma meter (Konica Minolta CR-400, Chuo-Ku, TYO, Japan) to determine the space color index L∗, a∗, b∗, C∗, and h∗. This device includes a designated area for placing each fruit and captures the color intensity through an active sensor following a manual activation. L∗ represents the lightness of the color and ranges from 0 (black) to 100 (white). a∗ represents the position between green and red. Negative values indicate green, while positive values indicate red. b∗ represents the position between blue and yellow. Negative values indicate blue, while positive values indicate yellow. C∗ represents the vividness or saturation of the color. It is calculated from the a∗ and b∗ values and describes how intense the color is. h∗ represents the color's hue, indicating its position on the color wheel. It is calculated from the a∗ and b∗ values and describes the type of color (e.g., red, yellow, green, blue) [24].

Afterward, the samples were subjected to destructive analysis for firmness and TSS measurements. Firmness was assessed using a fruit firmness tester (BioWorks FirmTech 500, Wamego, KS, USA). This device features a semi-perforated plate for placing the fruit sideways, which rotates automatically while an electric bar strikes each fruit individually from top to bottom to make the assessment. Finally, TSS analysis was performed using a refractometer (Atago PAL-1, Tokyo, Japan). Unlike the previous analyses, the 20-fruit samples were squeezed and homogenized for better sample representation. To ensure a comprehensive analysis, the measurements from the 20 individual fruits for each parameter were averaged. This approach provided a representative value for the entire sample for diameter, color, firmness, and TSS, reflecting the overall quality of the blueberries.

### 2.5 Data analysis and visualization

Data analysis and visualization are crucial for presenting research findings clearly and comprehensibly. To ensure that readers fully grasp the study's results, we carefully selected appropriate visual representations. This is particularly important in research involving complex methodologies, such as those incorporating image data and advanced statistical models, including AI-based approaches. Effective data visualization enhances understanding and also facilitates the communication of intricate information to a broader audience. Consequently, in this study, significant effort was dedicated to ensuring clarity and insight through the use of well-designed graphical representations. The following sections discuss the sequential data analysis methods employed, while specific packages and hyperparameters used for modeling are detailed in the Supplementary Table 1.

**Descriptive analysis:** We conducted a descriptive analysis to explore the temporal evolution and dispersion of the data from the images and laboratory analysis. This is visualized using boxplots, which provide an initial understanding of underlying trends and variability.

**Pearson's correlation analysis:** We conducted Pearson's correlation analysis to quantify the strength and direction of relationships between key parameters. The results are presented in a correlation matrix graph, offering a clear view of the interdependencies among variables.

**Bayesian generalized linear models (BGLM) for dataset simulation:** Recognizing the challenges posed by low variability in the dataset, we utilized BGLM to simulate a more robust dataset. This approach enabled us to enhance the dataset's comprehensiveness and its predictive accuracy. We simulated 4000 iterations using the Red, Green, and Blue values for TSS and firmness. The results are represented by line graphs. The average values from these simulations were then incorporated into predictive models.

**Predictive modeling with machine learning algorithms:** We split the dataset into 70 % for training and 30 % for testing, then eight machine learning (ML) algorithms were employed to develop predictive models: multiple linear regression (MLR), decision tree (DT), support vector machine (SVM), random forest (RF), extreme gradient boosting (XGB), k-nearest neighbor (KNN), artificial neural network (ANN), and partial least squared regression (PLSR). The performance of these models was assessed using key metrics: coefficient of determination (R^2^), mean absolute error (MAE), and root mean squared error (RMSE). The results are visualized using scatter plots, showcasing the dispersion of predicted versus observed values.

**Technical details:** All analyses were performed using the R programming language (version 4.4.0) within the RStudio environment (version 2024.04.2 + 764). The computations were executed on a desktop PC running Windows 11 Enterprise, equipped with an Intel® Core™ i9-14900K processor, 64 GB of RAM, a 2 TB SSD, and an NVIDIA GeForce RTX 4060.

## 3 Results

### 3.1 Temporal evolution and distribution of the blueberry fruit parameters

**Fig. 2:**
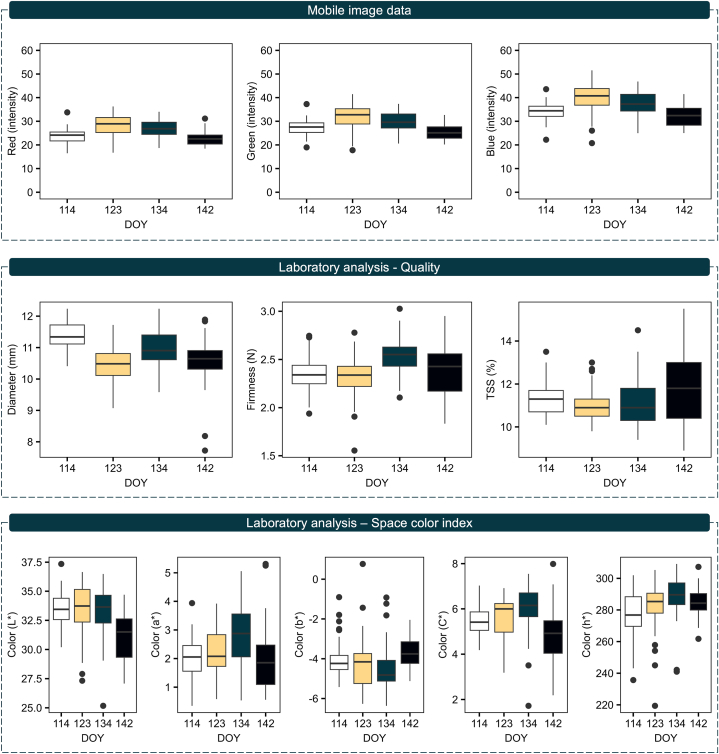
Boxplot representing the temporal evolution and variation of the dataset. The top section presents the image data (RGB intensity), the middle section presents the qualitative parameters (diameter, firmness, and TSS), and the bottom section presents the space color index analysis (L∗, a∗, b∗, C∗, and h∗). The y-axis represents the feature scale, and the x-axis represents the day of the year (DOY), which corresponds to the harvested day.

Our dataset encompasses image data (RGB intensity), qualitative parameters (diameter, firmness, and TSS), and space color index (L∗, a∗, b∗, C∗, and h∗). Notably, each feature exhibited distinct patterns corresponding to each harvest date (Fig. 2). Regarding the image data, the color intensity followed a polynomial trend: it was lowest at 114 DOY, peaked at 123 DOY, and subsequently declined. This pattern was consistent across all color bands. Overall, the fruits exhibited low color intensity, with a maximum value of 50 on a 0–255 scale, visually indicating how ripe the fruits were at each harvesting time. However, the intensity varied by band, with Red showing the lowest intensity, Green slightly higher, and Blue the highest.

Based on the quality analysis, notable results were found. The largest fruits were harvested at 114 DOY, their size decreased by 123 DOY, increased again by 134 DOY, and slightly decreased by 142 DOY. Firmness remained relatively stable between 114 and 123 DOY, increasing at 134 DOY, and showed greater variation at 142 DOY. Lastly, TSS levels were highest at 114 DOY, slightly decreased at 123 DOY, showed high variability at 134 DOY, and maintained high variation at 142 DOY, but with the highest values. Regarding the space color analysis, the results revealed the natural color characteristics of ripened blueberry fruits, with darkness (low L∗ and C∗ values), a tendency towards red and blue colors (positive a∗ values, negative b∗ values, and h∗ values mostly ranging from 270 to 290). However, variations were noted based on harvest date. For instance, the spaces color indices L∗, a∗, C∗, and h∗ values initially presented low values at 114 DOY, increased slightly until 134 DOY, and decreased by 142 DOY. Conversely, b∗ presented an opposite trend.

### 3.2 Correlating blueberry fruit color with its quality

**Fig. 3:**
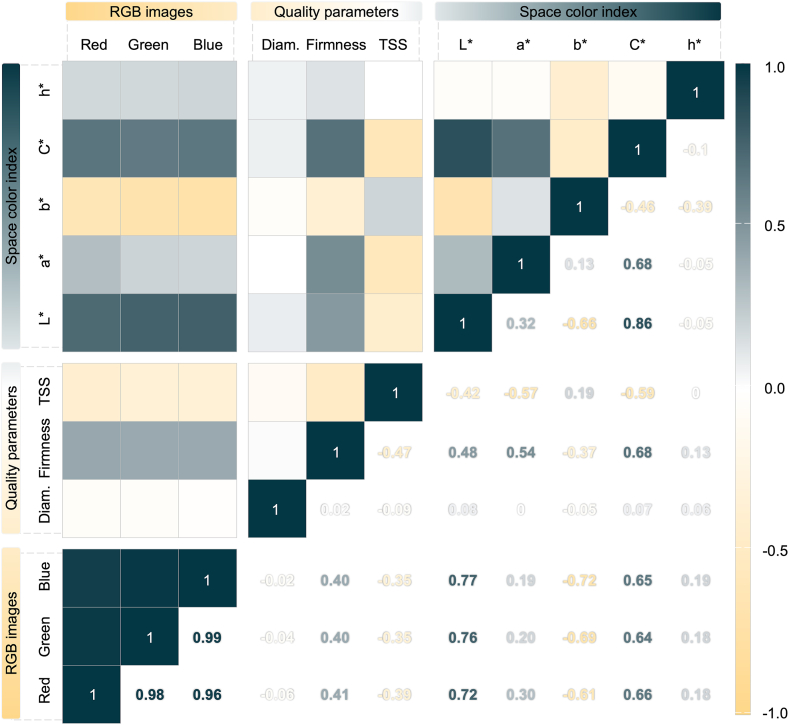
Pearson's correlation analysis between the image data (RGB intensity), qualitative parameters (diameter, firmness, and TSS), and space color indices (L∗, a∗, b∗, C∗, and h∗). The color gradient, transitioning from yellow to dark grey, indicates the strength of the correlation: yellow represents a high negative correlation (close to −1), white signifies a weak correlation (close to 0), and dark grey denotes a high positive correlation (close to 1).

Correlation analysis revealed an interesting relationship between the RGB images, space color indices, and fruit quality parameters (Fig. 3). Notably, the RGB data showed a very strong correlation among the RGB bands themselves (r > 0.96) and a strong correlation with some space color indices (L∗, b∗, and C∗) (r = 0.61–0.77). Regarding the qualitative parameters, the RGB colors demonstrated a moderate positive correlation with firmness (r < 0.41) and a moderate negative correlation with TSS (r < −0.39). Conversely, diameter produced a very low correlation (r < −0.06). Furthermore, the space color indices displayed strong correlations both among themselves and with the qualitative parameters, indicating that the color metrics are highly valuable for assessing fruit quality. Consequently, this correlation analysis produces timely and insightful results, identifying significant features for potential inclusion in predictive models for determining fruit quality.

### 3.3 Simulating blueberry dataset on BGLM and predicting fruit quality through ML-driven models

**Fig. 4:**
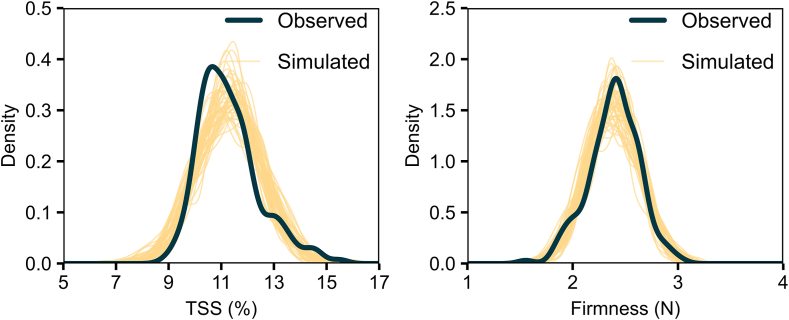
Graphical posterior predictive check comparing the observed distribution of TSS (left) and firmness (right) scores (dark line) with 4000 simulated datasets from the posterior predictive distribution (yellow lines).

Intriguing findings emerged from a graphical posterior predictive check comparing the observed distributions of TSS and firmness scores with simulated datasets generated from the BGLM (Fig. 4). The observed TSS distribution exhibited a pronounced peak of around 11 %, with a density approaching 0.4. The simulated datasets successfully captured the overall shape of this distribution, displaying similar peaks near the same TSS value, albeit with some variation in the spread and height of the density curves. For firmness, the observed data showed a peak around 2.5 N, with a density nearing 2.0. The simulated datasets closely mirrored the observed distribution, demonstrating strong alignment in both the peak and spread. Specifically, the observed and simulated datasets shared similarities in terms of average (TSS = 11.29 ± 0.001 %; Firmness = 2.39 ± 0.0004 N) and standard deviation (TSS = 0.84 ± 0.33 %; Firmness = 0.17 ± 0.069 N). Overall, the BGLM analysis produced simulations that closely align with the observed data, indicating that the model effectively captures the key features of the TSS and firmness distributions. The overlap between the simulated curves and the observed data suggests that the model performs well in replicating the observed patterns.

**Fig. 5:**
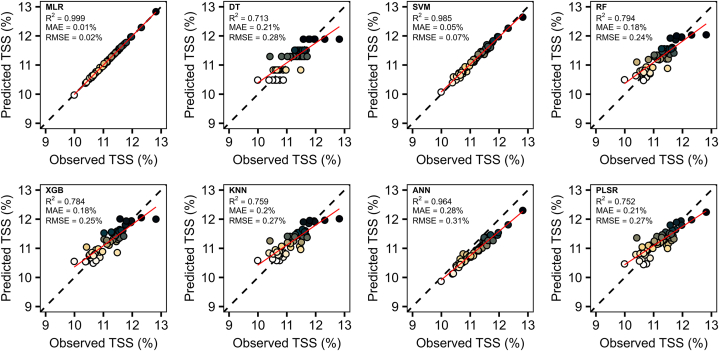
Predictive models for TSS. Predictive models were developed using the average simulated dataset from Bayesian analysis to estimate TSS. The following machine learning (ML) algorithms were used: multiple linear regression (MLR), decision tree (DT), support vector machine (SVM), random forest (RF), extreme gradient boosting (XGB), k-nearest neighbors (KNN), artificial neural network (ANN), and partial least squares regression (PLSR). The points' color gradient, transitioning from white to yellow to dark, indicates the range from low to high values, respectively.

**Fig. 6:**
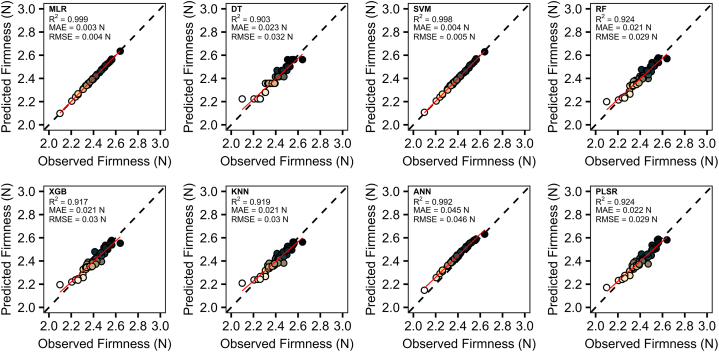
Predictive models for firmness. Predictive models were developed using the average simulated dataset from Bayesian analysis to estimate firmness. The following machine learning (ML) algorithms were used: multiple linear regression (MLR), decision tree (DT), support vector machine (SVM), random forest (RF), extreme gradient boosting (XGB), k-nearest neighbors (KNN), artificial neural network (ANN), and partial least squares regression (PLSR). The points' color gradient, transitioning from white to yellow to dark, indicates the range from low to high values, respectively.

The simulated dataset from BGLM was utilized to perform various predictive models. Notably, all the ML algorithms demonstrated strong predictive capabilities, achieving both high precision and accuracy for TSS (R^2^ = 0.71–0.99, MAE = 0.01–0.28 %, and RMSE = 0.02–0.31 %) (Fig. 5) and firmness (R^2^ = 0.90–0.99, MAE = 0.003–0.045 N, and RMSE = 0.004–0.046 N) (Fig. 6). For TSS, MLR provided the best fits, with R^2^ values approaching perfection. SVM and ANN also performed exceptionally well. Clearly, DT produced the lowest metrics values. In the case of firmness predictions, the majority of models exhibited strong performance (R^2^ > 0.90), with MLR, SVM, and ANN standing out, achieving R^2^ values exceeding 0.96.

**Fig. 7:**
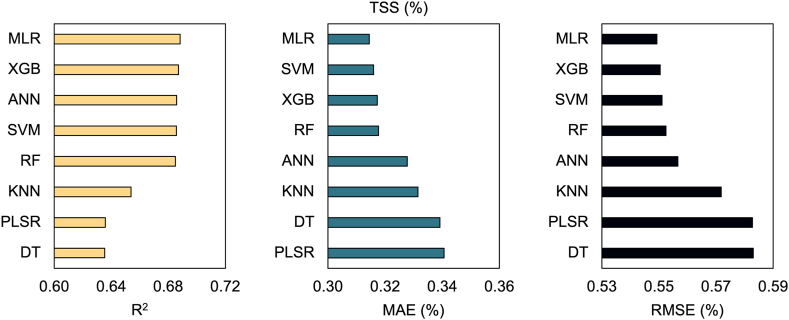
Predictive models for TSS. Predictive models were developed using the average simulated dataset from Bayesian analysis and applied to the observed dataset to estimate TSS. The following machine learning (ML) algorithms were used: multiple linear regression (MLR), decision tree (DT), support vector machine (SVM), random forest (RF), extreme gradient boosting (XGB), k-nearest neighbors (KNN), artificial neural network (ANN), and partial least squares regression (PLSR).

**Fig. 8:**
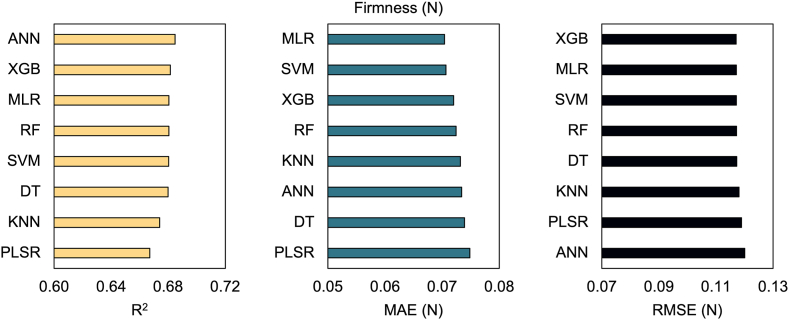
Predictive models for firmness. Predictive models were developed using the average simulated dataset from Bayesian analysis and applied to the observed dataset to estimate firmness. The following machine learning (ML) algorithms were used: multiple linear regression (MLR), decision tree (DT), support vector machine (SVM), random forest (RF), extreme gradient boosting (XGB), k-nearest neighbors (KNN), artificial neural network (ANN), and partial least squares regression (PLSR).

To further evaluate the models’ performance, we also tested them considering the original dataset. Interestingly, the models maintained their initial behavior, with linear- and tree-based models performing better. However, as expected, their effectiveness decreased for both TSS (R^2^ = 0.64–0.69, MAE = 0.31–0.34 N, and RMSE = 0.55–0.58 N) (Fig. 7) and firmness (R^2^ = 0.67–0.69, MAE = 0.070–0.075 N, and RMSE = 0.117–0.120 N) (Fig. 8).

## 4 Discussion

### 4.1 The nature of blueberry fruits ripening: implications for precision harvesting

The ripening of blueberry fruits is characterized by a series of physiological changes, including a shift in skin color from green to red and eventually to dark blue. Initially, green fruits exhibit high chlorophyll levels, which play a crucial role in the accumulation of anthocyanins [25]. As the fruit transitions from red to dark blue, chlorophyll levels decline and stabilize [11,26]. The dark blue coloration of the fruit's pericarp serves as a prominent indicator of full maturation, a traditional criterion for determining the optimal harvest time [11]. Harvesting is traditionally performed manually, a method that ensures superior fruit selection in terms of quality [27]. Manual harvesting allows for careful selection, minimizing the inclusion of under-ripe or damaged berries. However, when labor is scarce or when dealing with taller blueberry varieties, farms may employ mechanical harvesters [28]. While these machines enhance productivity and reduce labor requirements, they can compromise the quality of the harvesting. Even with optimal parameter settings, mechanical harvesting tends to result in a higher proportion of green, unripe berries being collected, while some ripe berries may remain on the plants. This limitation poses a challenge to maintaining the high-quality expected blueberry harvested fruits.

During the ripening process, blueberries primarily accumulate sucrose, fructose, and glucose—the three major soluble sugars—contributing to the fruit's sweetness [29]. The sweetness of the fruit is generally associated with a high TSS content, which is considered a desirable trait. However, it is important to note that sweetness levels in blueberry fruits vary significantly during different stages of maturation (Fig. 2), particularly between stages S3 and S4 [26]. Previous studies have documented TSS values ranging from 8 % to 15 % during the maturity stages [30,31]. In addition to sugar content, fruit firmness is a critical factor in determining the appropriate harvest time. Firmness is essential for ensuring the fruit's resistance to damage during harvest, handling, storage, and shelf life [32]. However, it is a natural phenomenon that as blueberries mature and accumulate sugars, their firmness tends to decrease [33], as noted in our results (Fig. 3). This reduction in firmness is influenced by various factors, including changes in cellular organelles, biochemical constituents, water content, and cell wall composition that occur during growth, development, or postharvest storage [34,35]. Similar to TSS, fruit firmness varies throughout the ripening process (Fig. 2), with values reported to range from 1 to 3 N in previous studies [26,34].

An understanding of the interaction between TSS and firmness levels is crucial for making informed harvest decisions. It is important to prioritize each parameter individually or in combination, depending on the specific industry requirements. The negative correlation between TSS and firmness is noteworthy. Sweeter fruits are often preferred for processing purposes, while firmer fruits are essential for fresh consumption and longer shelf life. However, making harvest decisions based on these parameters is complex, particularly when relying on traditional methods. Factors such as weather conditions, varieties, and regional differences can further complicate the decision-making process. However, by examining the interactions between TSS and firmness, growers can more accurately determine the optimal harvest time for different market segments. For instance, firmness reached its peak value at 134 days of the year (DOY), indicating an optimal harvest date for market-bound produce. Conversely, higher TSS values were observed at 142 DOY, making this the preferred date for harvesting fruit intended for processing. These considerations extend beyond merely determining the harvest date; they also inform spatial delineation strategies, providing a basis for making informed decisions regarding harvest locations.

### 4.2 Images as a suitable data source for fruit quality assessment

Recent studies have increasingly leveraged images as a primary source of data for decision-making in crop harvesting. The use of imagery presents a novel opportunity to bypass the subjectivity inherent in traditional methods, such as visual inspections with the naked eye [36,37] or labor-intensive laboratory analyses [38]. This approach significantly reduces costs and time while providing more representative data at larger scales. However, the effective use of images demands a high level of technological expertise and substantial resources for data processing. Images are not merely visual representations; they are rich sources of quantitative information. Therefore, effectively handling this data is crucial and highlights the importance of integrating advanced computational technologies, which significantly contribute to this process. Many previous studies have successfully utilized images for fruit detection and yield prediction, directly aiding in harvest activities for various crops such as citrus [39], *Camellia oleifera* [40], and cotton [41]. However, extending image analysis to evaluate not only yield but also fruit quality marks a significant advancement in agricultural technology. This progress will enable more precise decision-making for both harvest and post-harvest processes.

As previously discussed, blueberry fruit quality is normally evaluated based on its color, making imagery a promising tool for quality assessment. In our study, for instance, we leveraged a dataset of blueberry fruit images to evaluate fruit quality. Notably, all images were meticulously collected from fully ripe fruits, which displayed subtle variations in color—differences that are typically undetectable by the human eye. These slight variations often complicate decision-making when relying solely on human judgment. However, through image analysis and advanced data processing techniques, we were able to detect even the most nuanced differences in these quality parameters. While several in-situ studies successfully utilized images to detect blueberry fruits for yield prediction and maturity estimation [14,16,42], our study goes a step further by predicting not only TSS and firmness but also doing so under challenging conditions where the fruits were ripened with minimal variation. This advancement highlights the potential of image-based assessments to enhance precision in quality evaluation, even in scenarios where traditional methods can fall short; therefore, offering a more objective and reliable approach to fruit quality evaluation.

### 4.3 ML-driven analysis for unveiling blueberry fruit quality through images

One significant observation from our analysis is the low variability within the dataset (Fig. 2), which presents a challenge for developing accurate predictive models. Despite this, our correlation analysis revealed moderate associations between image data and qualitative fruit parameters (r < 0.41) (Fig. 3). While this suggests a promising trend for the application of imaging techniques, the correlations are not always strong enough to make definitive decisions. Consequently, we followed a more in-depth analysis of the data. Specifically, we employed a BGLM to account for prediction uncertainty by generating a distribution of possible outcomes, rather than relying on a single deterministic result. As a result, this approach simulated a dataset with very similar characteristics to the observed data, ensuring the reliability of our findings (Fig. 4). This approach leverages the complementary strengths of various models to produce more reliable ensemble results and mitigate random errors [43]. It is also applied for small sample sizes, especially likelihood-based inference can be unreliable with variance components being particularly difficult to estimate [44].

Subsequently, we applied eight ML algorithms to develop predictive models. Among these, MLR and SVM consistently outperformed other models in terms of R^2^, MAE, and RMSE, demonstrating their effectiveness in predicting both TSS and firmness (Fig. 5, Fig. 6). Linear-based models can perform well on datasets that exhibit high linearity, as supported by previous independent studies [[45], [46], [47]]. However, it is crucial to note that despite the superior results of linear models, this does not imply they should be used exclusively. Their simplicity and ease of interpretation come at the cost of limited ability to handle non-linearity [48,49]. Additionally, it is noteworthy that our dataset underwent linear simulation through BGLM, which certainly favored the better performance of linear models. Consequently, incorporating more sophisticated ML algorithms can enhance model generalizability and robustness. For example, the ANN model also showed strong performance for both variables. This particular algorithm presents high data analysis capabilities and typically exhibits outstanding performance [50]. In addition to handling complex nonlinear problems [51], this model prevents overfitting and underfitting [52]. Furthermore, RF and XGB presented timely results. In general, tree-based models have been extensively used in the literature due to their capability to handle complex and robust datasets. RF and XGB, for instance, are composed of multiple decision trees, which generally produce robust and accurate models [53]. However, when applied to a limited dataset, the complexity of these models poses challenges in tuning the hyperparameters, leading to suboptimal performance [54]. Moreover, KNN, PLSR, and DT produced good results but were less accurate. KNN, in particular, is a simple ML algorithm that considers neighbors' observations when defining the weights, with closer points contributing more [55]. Previous studies have highlighted that its high performance in predictive approaches is dependent on a high correlation with the dependent variable and the presence of low multicollinearity, which could make KNN more sensitive, for example, than RF [56]. The superior performance of KNN has also been reported when compared with XGB [57]. Additionally, PLSR has been questioned regarding the number of observations and explanatory variables. A higher number of inputs has demonstrated challenges to the model's performance, especially if the number of observations is smaller than the number of inputs [58,59]. Ultimately, DT produced less effective results. Although DT is known for its interpretability, its learning process is challenging and experiences overfitting when the models become more complex, consequently producing poor results [53].

This study highlights the significance of ML models in predictive analysis, as the choice of model can significantly impact performance depending on the nature of the data and the response variable. Future research should further investigate the data characteristics that contribute to models’ effectiveness and explore potential optimizations for enhancing predictive accuracy. For example, integrating multiple ML algorithms into a single model to enhance the effectiveness [60]. Importantly, our findings demonstrate that we can effectively predict both TSS and firmness using a variety of ML algorithms, validating our approach and paving the way for future research in this area. Moreover, this study advances the field by employing algorithms that utilize less computational power and offer greater interpretability than previously DL-based methodologies [[16], [17], [18], [19]], yet deliver enhanced results.

### 4.4 Advancing scientific understanding and guiding future research

#### 4.4.1 The value of our study

The determination of blueberry fruit quality using image analysis represents a cutting-edge advancement in agricultural technology. Our study is pivotal in designing a robust framework that facilitates informed decision-making, thereby advancing the agricultural sector. Traditionally, blueberry harvest decisions have been based on visual inspection and in-situ selection, which is completely subjective. This study, however, highlights the practical application of mobile devices for capturing RGB images that can accurately assess blueberry fruit quality. When combined with strategic ML-driven data analysis, these images pave the way for the development of highly accurate predictive models—previously achievable only through labor-intensive and costly laboratory equipment. Furthermore, integrating our approach with blueberry yield prediction [61,62], in-situ fruit detection techniques directly on the plant [16], models for rapid detection of mechanical damage [63], and high-throughput phenotyping (HTP) [64] can substantially enhance the efficiency and precision of the blueberry assessment and support harvesting process. Notably, our findings contribute to the technological advancement of blueberry production, making it more competitive within the specialty crops sector [65], particularly in the context of harvest technology [66].

#### 4.4.2 Limitations, future studies, and contributions

This research, to the best of our knowledge, represents the first study to use mobile images to assess blueberry fruit quality (TSS and firmness). Consequently, limitations arise. Initially, our dataset is relatively small compared to typical agricultural datasets (e.g., yield data). However, the labor-intensive data collection process, the delicate nature of the blueberry fruit, and the meticulous laboratory analyses required to ensure sample integrity limited the sample size. Additionally, laboratory-based imaging was chosen to ensure consistency and reproducibility during initial model development and to establish whether such images could effectively predict fruit quality. Having demonstrated the feasibility of this approach, future studies will include images captured under real-world conditions. Furthermore, we employed BGLM to simulate additional data, allowing us to develop more robust models. However, when applying the models to the real dataset, the performance was reduced, a common and expected occurrence in ML when transitioning from controlled to real-world conditions. Conversely, the results remain very important. They highlight both the potential of the approach and the challenges of models’ transferability and generalizability. Certainly, our approach likely ensures reliable results under similar conditions. However, we recognize that changes in the environment, fruit patterns, and different devices can limit the immediate applicability of our approach. While these limitations are inherent to a pilot study, they can be readily considered in future studies without compromising the validity of our current findings. Therefore, future investigations will focus on enhancing our method by developing a more robust dataset, which includes data from additional years, varieties, and samples, as well as establishing device standards.

Despite the aforementioned limitations, our study provides crucial insights for the scientific community. It introduces the unprecedented approach of mobile-based RGB images and ML algorithms for assessing blueberry fruit quality. It establishes a foundation for both immediate and future harvest decision-making processes and supports the development of research proposals involving laboratory analysis. Below, we outline the significant contributions of our study:

i. **Development of real-time mobile applications:** The versatility of our predictive model extends to the development of mobile applications for real-time blueberry quality assessment. Such an application would allow users to take a picture of the fruit and receive immediate quality information. This tool could be particularly valuable for both field and laboratory conditions. In the field, farmers could use the application to make quick, informed decisions about harvest timing and fruit sorting. In laboratory settings, the application could streamline quality assessments by providing instant, objective measurements of fruit attributes.
ii. **Support for laboratory research and breeding programs:** By enabling rapid and non-destructive assessments of fruit quality, our model facilitates the evaluation of large sample sizes, thereby accelerating the selection of desirable traits in breeding programs.
iii. **Application in mechanical harvesting:** By embedding our model into the harvester's system, it becomes possible to classify fruit immediately after picking. This ensures that only high-quality fruit is stored, reducing the post-harvest sorting burden and improving overall product quality. Although this classification occurs post-harvest, it significantly streamlines subsequent labor tasks and enhances the efficiency of the harvest process.
iv. **Integration with emerging agricultural robotics:** These platforms could perform real-time recognition and classification of blueberries, enabling autonomous harvesting decisions based on key quality metrics such as TSS and firmness. This integration would enhance the efficiency of harvesting operations by reducing human error, labor costs, and maximizing yield quality.

## 5 Conclusion

In this study, we aimed to address the challenges of traditional blueberry fruit quality assessment methods, which are often labor-intensive, time-consuming, and costly. To achieve this, we proposed an innovative approach using mobile image-based and ML-driven algorithms. Our analysis demonstrated that mobile images significantly contributed to assessing TSS and firmness; however, the integration of ML algorithms enhanced the predictive accuracy of our models. Notably, our predictive models were effective, achieving high precision (high R^2^) and high accuracy (low MAE and RMSE). This innovative approach contributes to the scientific understanding of image-based quality evaluation while potentially paving the way for future advancements in agricultural technology. Furthermore, despite these promising results, we also recognize that our approach can be further enhanced by incorporating data from diverse environments and varieties, and by establishing standards for mobile device usage. These improvements will significantly contribute to the practical application of our approach.

## CRediT authorship contribution statement

**Marcelo Rodrigues Barbosa Júnior:** Conceptualization, Data curation, Formal analysis, Investigation, Methodology, Validation, Visualization, Writing – original draft, Writing – review & editing. **Regimar Garcia dos Santos:** Investigation, Validation, Visualization, Writing – review & editing. **Lucas de Azevedo Sales:** Investigation, Validation, Visualization, Writing – review & editing. **Rônega Boa Sorte Vargas:** Investigation, Validation, Visualization, Writing – review & editing. **Angelos Deltsidis:** Resources, Visualization, Writing – review & editing. **Luan Pereira de Oliveira:** Conceptualization, Funding acquisition, Investigation, Methodology, Project administration, Supervision, Validation, Visualization, Writing – review & editing.

## Declaration of generative AI and AI-assisted technologies in the writing process

During the preparation of this work, the authors utilized Microsoft Copilot, an AI-assisted technology, to improve the overall readability of the manuscript. After utilizing this tool/service, the authors reviewed and edited the content as necessary. As a result, they take full responsibility for the accuracy, integrity, and scientific rigor of the publication.

## Funding

This work was supported by the Georgia Agricultural Commodity Commission for Blueberries.

## Declaration of competing interest

The authors declare that they have no known competing financial interests or personal relationships that could have appeared to influence the work reported in this manuscript.
