# Supplementary material for: Image-based and ML-driven analysis for assessing blueberry fruit quality
Source: Heliyon. 2025 Jan 27;11(3):e42288. doi: 10.1016/j.heliyon.2025.e42288 (PMC11834081; doi:10.1016/j.heliyon.2025.e42288)
Supplement: Multimedia component 1 [file mmc1.pdf]

Supplementary Table 1. Machine learning algorithms and R packages used in this study.

Algorithm name	Abbreviation	Formula	Package	Reference
Multiple linear regression	MLR	lm(y ~ Red + Green + Blue, dataset)	stats	(Wilkinson and Rogers, 1973)
Decision tree	DT	rpart(y ~ Red + Green + Blue, dataset)	rpart	(Breiman, 2017)
Support vector machine	SVM	svm(y ~ Red + Green + Blue, dataset, type = "nu-regression", kernel = "linear")	e1071	(Fan et al., 2005)
Random forest	RF	randomForest(y ~ Red + Green + Blue, dataset, mtry = 3, ntree = 1000)	randomForest	(Breiman, 2001)
Extreme gradient boosting	XGB	xgb.DMatrix(y ~ Red + Green + Blue, dataset)<br>params <- list(objective = "reg:squarederror", booster = "gbtree", eta = 0.1, max_depth = 6, subsample = 0.8, colsample_bytree = 0.8)<br>xgb_brix <- xgb.train(params = params, data = dtrain, nrounds = 1000)	xgboost	(Chen and Guestrin, 2016)
K-nearest neighbor	KNN	knnreg(y ~ Red + Green + Blue, dataset, k = 5)	caret	(Ripley, 2007)
Artificial neural network	ANN	neuralnet(y, Red + Green + Blue, dataset, hidden = c(5, 3), linear.output = TRUE)	neuralnet	(Intrator and Intrator, 1993)
Partial least squared regression	PLSR	plsr(y ~ Red + Green + Blue, dataset, scale = TRUE, validation = "CV")	pls	(Martens and Næs, 1992)

All formulas include *y*, which was replaced by brix and firmness individually during the predictions. Additionally, *dataset* refers to the training dataset, which accounts for 70% of the original dataset, while the testing dataset accounts for 30%.
